# Phasic activation of the locus coeruleus attenuates the acoustic startle response by increasing cortical arousal

**DOI:** 10.1038/s41598-020-80703-5

**Published:** 2021-01-14

**Authors:** Mingyu Yang, Nikos K. Logothetis, Oxana Eschenko

**Affiliations:** 1 Department of Physiology of Cognitive Processes, Max Planck Institute for Biological Cybernetics, Tübingen, Germany; 2 Division of Imaging Science and Biomedical Engineering, University of Manchester, Manchester, M13 9PT UK

**Keywords:** Sensorimotor processing, Reflexes, Neuroscience, Auditory system, Midbrain

## Abstract

An alerting sound elicits the Acoustic Startle Response (ASR) that is dependent on the sound volume and organisms’ state, which is regulated by neuromodulatory centers. The locus coeruleus (LC) neurons respond to salient stimuli and noradrenaline release affects sensory processing, including auditory. The LC hyperactivity is detrimental for sensorimotor gating. We report here that priming microstimulation of the LC (100-ms at 20, 50, and 100 Hz) attenuated the ASR in rats. The ASR reduction scaled with frequency and 100 Hz-stimulation mimicked pre-exposure to a non-startling tone (prepulse). A rapid (~ 40 ms) EEG desynchronization following the LC stimulation suggested that the ASR reduction was due to elevated cortical arousal. The effects of LC stimulation on the ASR and EEG were consistent with systematic relationships between the ASR, awake/sleep state, and the cortical arousal level; for that matter, a lower ASR amplitude corresponded to a higher arousal level. Thus, the LC appears to modulate the ASR circuit via its diffuse ascending projections to the forebrain saliency network. The LC modulation directly in the brainstem and/or spinal cord may also play a role. Our findings suggest the LC as a part of the brain circuitry regulating the ASR, while underlying neurophysiological mechanisms require further investigation.

## Introduction

An alerting stimulus can induce an eye blink, contraction of the facial, neck, and skeletal muscles as well as various visceral reactions. These innate startle reflexes, which are triggered by abrupt environmental changes, initiate more complex forms of adaptive response from orienting and exploration^1^ to defensive behaviors^2^. The startle reflex can be elicited through different sensory modalities, including visual, tactile, vestibular, or acoustic. The Acoustic Startle Response is known to depend on the sound volume, but also the arousal level or emotional state^3^. The ASR is attenuated by pre-exposure to a non-startling stimulus (prepulse). The ASR and prepulse inhibition (PPI) are commonly used for testing sensorimotor integration in animals and humans and as diagnostic tools for mental fatigue and various neuropsychiatric conditions with disrupted sensorimotor processing, such as schizophrenia, attention disorder, or autism^4,5^. The primary mammalian ASR circuit consists of a short pathway linking the auditory nerve and cochlear root neurons (CRN) with spinal motor neurons through the caudal pontine reticular nucleus (PnC)^6–8^. The PPI circuit is more complex and the mechanisms mediating PPI are not yet fully understood^9,10^. It is commonly accepted that the auditory prepulse is relayed through the CRN, the inferior (IC) and superior (SC) colliculi, and activates the pedunculopontine tegmental nucleus (PPTg), which sends inhibitory projections on the PnC giant neurons resulting in the ASR attenuation^3,9,10^. Despite the existence of multiple top-down and neuromodulatory inputs converging on the PnC^11^, the predominant role for inhibiting the ASR circuit has been long assigned to the cholinergic projection from the PPTg to PnC^3^. However, new evidence challenged this long-standing view^12–14^ encouraging reconsidering the functional connectivity of the ASR/PPI circuit.

The brainstem noradrenergic nucleus locus coeruleus is a part of the ascending arousal system^15^. The role of LC phasic response in mediating the orienting response and attention is well known^16^. Salient stimuli elicit phasic discharge of LC neurons^17,18^ and associated noradrenaline (NA) release in the LC forebrain targets affects sensory processing^19,20^, including auditory^21–23^. The results of pharmacological and lesion studies suggested that the LC-NA system exerts an excitatory effect on the ASR circuit^24–27^. The LC hyperactivity and enhanced NA transmission within distinct thalamocortical and ventral forebrain networks lead to the PPI deficiency^28,29^. In contrast, the LC phasic response promotes cortical encoding of salient stimuli^30^. It has been long established that elevated tonic firing of the LC-NA neurons makes the LC sensory-evoked response less pronounced^31^; the latter possibly results in less efficient sensorimotor integration. To our knowledge, LC neural activity has not been characterized using the ASR/PPI paradigm, leaving the role of the LC auditory-evoked response in the sensorimotor gating underlying ASR and PPI unknown. In the present study, we mimicked the LC phasic response by applying a mild electric current to the LC cell bodies^32^ and paired the LC stimulation with the startling sound. In the past, microstimulation proved to be a valuable tool for dissecting the ASR/PPI circuit. Earlier studies applied electrical stimulation to the PPTg, IC, SC, or the ventral pallidum to simulate prepulse^33^. In Parkinson's disease patients, microstimulation of the subthalamic nucleus reduced the ASR^34^. Here we report that phasic LC activation paired with a startle-eliciting sound attenuated the ASR to the same extent as the auditory prepulse.

## Results

In total, 21 adult male rats were used in this study. Each rat was first habituated to the test chamber where rats were exposed to sounds and the electroencephalography (EEG) recording and microstimulation took place. The rat motor activity level was assessed by measuring the deflection amplitude of a movement-sensitive floor. Before the main experiment, each rat was subjected to preliminary tests aiming at adjusting the acoustic and microstimulation parameters. In all rats, the intensity of prepulse sound was adjusted not to elicit any above-threshold movement, whereas the startling sound was set to reliably evoke the ASR. In the first cohort of rats (n = 14), two skull screws were used for EEG monitoring and a stimulating electrode was implanted in the LC area. After post-surgical recovery rats were exposed to acoustic stimuli (prepulse: 10 kHz pure tone, 20 ms, 70 dB; startle: broadband noise, 40 ms, 100 dB) presented separately or sequentially (prepulse followed by startle) with a 100-ms interval in random order with a 10–20 s inter-trial interval (ITI). In the vast majority of the prepulse trials (range: 90–100%), the floor deflection amplitude remained within the 95% confidence interval (CI) calculated during episodes when no auditory stimuli were presented. The startling sound elicited above-threshold movement in 100% of trials in all rats. In the paired trials, when the startling sound was preceded by the prepulse, the ASR amplitude was consistently attenuated (Kolmogorov–Smirnov test, p < 0.05 for all rats). This phenomenon is commonly referred to as PPI. The average ASR reduction was 73.1 ± 2.4%.

**Figure 1:**
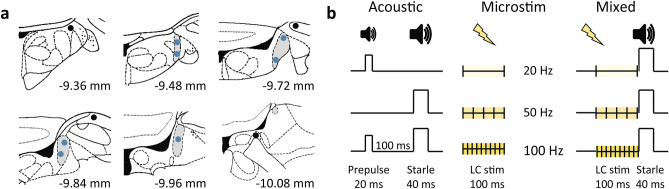
Experimental design. (**a**) Placement reconstruction of the stimulation electrode. The LC core is shown in grey; filled circles show the electrode tips. Different anterior–posterior planes are shown; numbers indicate the distance from bregma according to the rat brain atlas^84^. (**b**) Schematic representation of the experimental design. Each session included acoustic, microstimulation, and mixed (microstimulation preceded by startle) trials resulting in 9 trial types. The acoustic trials included prepulse, startle alone or preceded by prepulse. The microstimulation trials included delivery of a mild (0.05 mA) electric current at different pulse frequencies (20, 50, and 100 Hz) for 100 ms. During mixed trials, microstimulation preceded the startling sound. Forty repetitions of each trial type were randomly presented with a 10–20-s ITI.

We next evaluated the effectiveness of microstimulation by the presence of a transient change in the EEG spectrogram. We have previously shown that the LC microstimulation in naturally sleeping rats caused an EEG power decrease in the delta (1–4 Hz) and sigma (12–16 Hz) bands and a power increase of frequencies above 30 Hz^35^. One rat had an invalid EEG signal and was excluded from the study. The ‘effective’ stimulation was observed in 7 out of 13 rats. Histological examination confirmed the electrode placement in the LC (Fig. 1a). The parameters for the LC stimulation have been further adjusted such that the strongest stimulation did not cause a motor response, awakening from sleep, or any aversive behaviors. The stimulation parameters were comparable to the ones used in our previous study^35^. In 6 rats, the application of electric current was ‘ineffective’ at any stimulation intensity. In 4 out of 6 rats, the electrode was located outside the LC (Fig. 1a); these rats made up a control (outside LC stimulation) group. In 2 rats, the electrode was in the LC (not shown); however, microstimulation was unreliable, likely due to the electrode damage or high impedance. Therefore, these 2 rats were excluded from the study.

The second cohort of rats (n = 7) was used for the assessment of sensorimotor gating as a function of arousal. The EEG was monitored, but no microstimulation was applied in this group. Preliminary testing using the same sound parameters as specified above showed that the ASR amplitude was mildly or not affected by preceding prepulse (Kolmogorov–Smirnov test, p > 0.05 for each rat). The average of ASR reduction was 21.6 ± 2.0% (n = 7 rats). To obtain a reliable PPI, we adjusted the sound parameters after a series of calibration trials. In 3 rats, the combination of 75 dB-prepulse and 100 dB-startle was optimal, whereas in other 4 rats a combination of 70 dB-prepulse and 105 dB-startle produced the most consistent PPI. Using the adjusted sound parameters, the startling sound elicited the ASR in the vast majority of trials (94.8 ± 1.6%; n = 7 rats), whereas prepulse did not induce any above-threshold movement in 94.3 ± 1.7% of trials. The ASR amplitude was consistently attenuated by the prepulse (Kolmogorov–Smirnov test, p < 0.05 for each rat). Despite a slight difference in the auditory parameters, %PPI was comparable across experimental conditions (Table 1).

**Table 1:** Experimental groups and sound parameters.

Experiment	Stimulation	Measurements	N rats (N sessions)	Sound level, dB prepulse/startle	PPI (%)*
Effect of LC phasic response on ASR	In LC	EEG, movement	7 (14)	70/100	70.8 ± 3.2
	Outside LC	EEG, movement	4 (14)		
Effect of arousal on ASR, PPI, and AEP	–	EEG, movement	3 (8)	75/100	74.4 ± 3.6
			4 (12)	70/105	78.5 ± 3.2

*Mean ± s.e.m are shown; %PPI did not differ between groups (one-way ANOVA, F_2, 47_ = 1.1, p = 0.3).

The data obtained from 18 rats were further analyzed (Table 1). Figure 1b illustrates the experimental design for the first cohort (n = 11). Each session included acoustic, microstimulation, and mixed (microstimulation preceded by startle) trials resulting in 9 trial types. The acoustic trials included prepulse, startle alone or preceded by prepulse. The microstimulation trials included delivery of a mild (0.05 mA) electric current at different pulse frequencies (20, 50, and 100 Hz) for 100 ms. During mixed trials, microstimulation preceded the startling sound. The second cohort (n = 7) was exposed to the acoustic trials only. In each session, 40 to 80 repetitions of each trial type were presented in random order with a 10–20 s ITI. The frontal EEG and rat’s motor activity were continuously monitored.

### Effect of priming LC stimulation on the acoustic startle response

**Figure 2:**
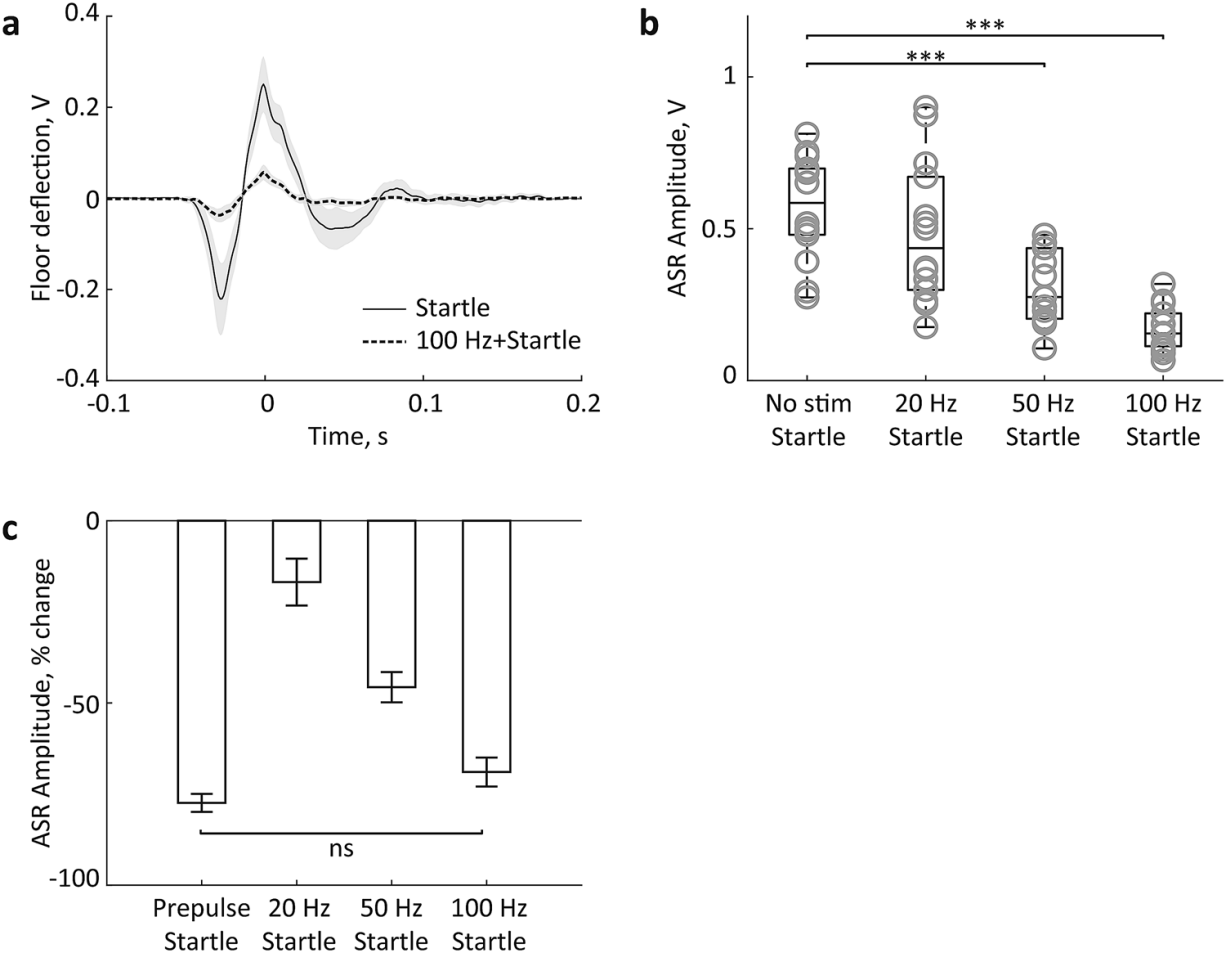
Priming LC stimulation attenuates the acoustic startle response. (**a**) The deflection shape of a movement-sensitive floor. Data from a representative session are shown. Traces are aligned to the maximal amplitude (t = 0) and averaged over the startle only trials (n = 40 trials) and the startle preceded by LC stimulation (n = 40 trials, 0.05 mA, 100 ms at 100 Hz); shadows show s.e.m. (**b**) The ASR amplitude elicited by the startle alone and the startle followed by LC stimulation at different frequencies. Box-whisker plots show the median, the first and third quartiles, min/max. Circles represent session averages. (**c**) The ASR amplitude change for different trial types. Note, the LC stimulation at 100 Hz mimicked the auditory prepulse. ***p < 0.001 (post hoc comparisons, Bonferroni corrected).

To examine the role of LC phasic activation for the ASR, we applied a brief, 100-ms train of electric pulses in the direct proximity to the cell bodies of LC neurons 100 ms before presentation of a startling auditory tone (see “Materials and methods”). The ASR was measured by the deflection of a motion-sensitive floor. The effect of LC stimulation was validated in 14 sessions (n = 7 rats, 1–3 sessions per rat); stimulation outside the LC was assessed in 14 sessions (n = 4 rats, 1–5 sessions per rat). The ASR amplitude was substantially reduced when the startling sound was preceded by the LC stimulation. Figure 2a illustrates the data from a representative session. The ASR reduction was consistent across sessions (Wilcoxon signed-rank test, Z = 3.3, p = 0.001, r = 0.9). The repeated-measures analysis of variance (ANOVA) revealed the main effect of the stimulation frequency (20, 50, and 100 Hz) on the ASR amplitude (F_1.1, 14.8_ = 24.5, p = 0.00012, eta^2^ = 0.7; Greenhouse–Geisser corrected). Paired comparisons showed that LC stimulation at 50 and 100 Hz, but not at 20 Hz, significantly reduced the ASR (Fig. 2b). Furthermore, the effect scaled with the stimulation frequency such as the LC stimulation at 100 Hz caused the same degree of the ASR attenuation as the one produced by a prepulse sound (Wilcoxon signed-rank test, Z = 1.4, p = 0.16, r = 0.4; Fig. 2c). Importantly, the LC stimulation alone did not elicit a detectable motor response at any stimulation frequency as reflected by the floor deflections remaining within the 95% CIs for all trials. Stimulation outside the LC paired with the startle sound did not reduce the ASR (Wilcoxon signed-rank test, Z = 1.0, p = 0.3, r = 0.3).

**Figure 3:**
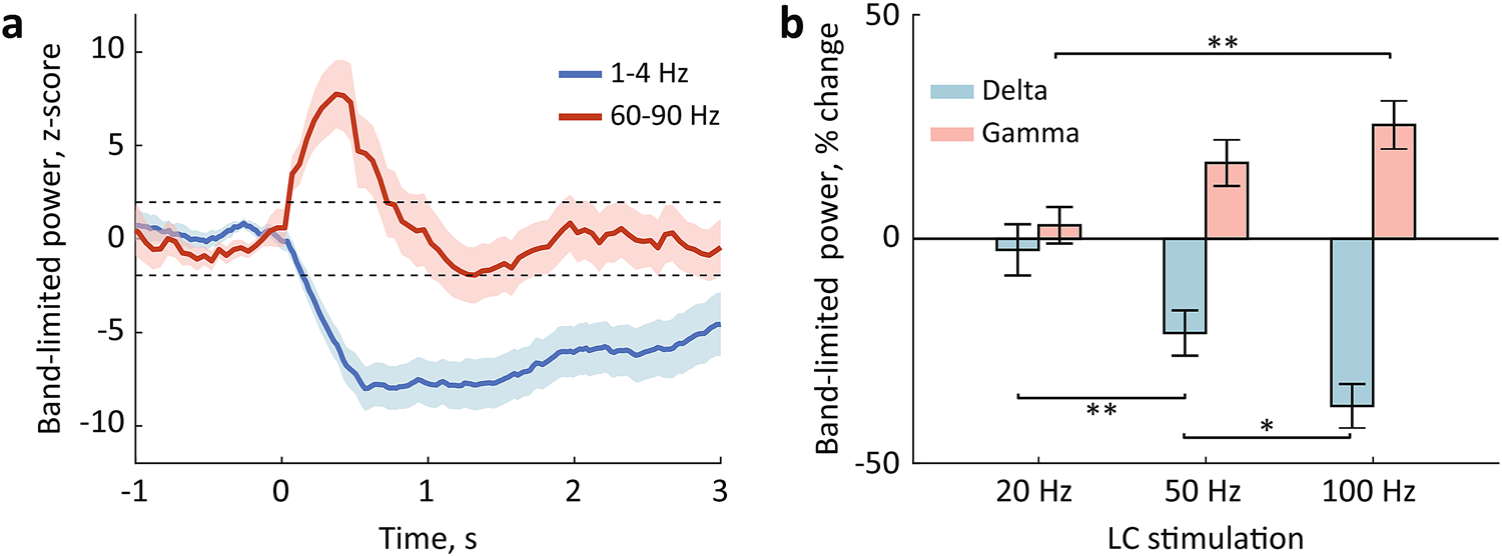
The EEG modulation by LC stimulation. (**a**) The EEG delta (1–4 Hz) and gamma (60–90 Hz) power around LC stimulation. Averages over 14 sessions (n = 7 rats) are plotted for LC stimulation at 100 Hz; shadows show s.e.m. (**b**) The delta and gamma power change produced by LC stimulation. *p < 0.05, **p < 0.01 (post hoc comparisons, Bonferroni corrected).

The LC phasic activation may have transiently affected the functional connectivity within the ASR circuit and resulted in a stronger inhibition of the PnC giant neurons; the latter could occur due to non-specific activation of the ascending arousal system. We have previously reported that LC phasic activation is followed by a cortical state change^32,35,36^. To gain insights on the mechanism underlying the ASR attenuation by LC phasic activation, we performed a spectral analysis of the frontal EEG. Consistent with previous studies, the LC stimulation resulted in a rapid (~ 40 ms) change in the EEG spectral composition. Specifically, delta (1–4 Hz) power transiently decreased, while the power of higher frequencies, including the high-gamma (60–90 Hz) range, increased (Fig. 3a). We used the high-gamma range to avoid the EEG artifacts caused by the electric pulses at 50 Hz. The pattern of the EEG modulation was indicative of cortical arousal. To quantify the EEG modulation, we extracted the band-limited power in the delta and gamma ranges and z-score normalized to a 1-s window preceding the LC stimulation onset. The change of both delta and gamma power exceeded the threshold (± 1.96 z-score) for all sessions. We then compared the degree of power change as a function of stimulation strength. The repeated-measures ANOVA revealed the main effect of the stimulation frequency for both delta (F_2, 26_ = 17.3, p < 0.0001, eta^2^ = 0.6) and gamma (F_2, 26_ = 8.8, p = 0.001, eta^2^ = 0.4) bands with the maximal EEG modulation produced by the LC stimulation at 100 Hz (Fig. 3b). Interestingly, LC stimulation at 20 Hz was inefficient for both EEG and ASR modulation.

As described above, the LC phasic activation was accompanied by a transient change in the ongoing cortical state, which was reminiscent of microarousal. The LC stimulation repeated multiple times during the ~ 90-min session and thus may have also caused a shift in the rat behavioral state. We classified rat behavior into active awake, quiet awake, or non-rapid eye movement (NREM) sleep (see “Materials and methods”) and compared across the LC-stimulated and control rats (outside-LC stimulation). Table 2 shows the percentage of time spent in each behavioral state. There was a significant effect of the behavioral state (F_2,52_ = 4.1, p = 0.02, eta^2^ = 0.1), but no significant state × group interaction (F_2,52_ = 1.2, p = 0.3, eta^2^ = 0.05). Thus, rats spent slightly less time in the active awake state; however, the LC stimulation did not affect rats’ behavioral pattern.

**Table 2:** The awake/sleep states during testing.

	N rats (N sessions)	Active awake	Quiet awake	NREM sleep
In-LC stimulation	7 (14)	24.0 ± 3.4	44.6 ± 3.7	31.0 ± 4.0
Outside-LC stimulation	4 (14)	32.3 ± 4.2	37.7 ± 3.0	30.0 ± 5.2
Combined	11 (28)	28.1 ± 2.7*	41.2 ± 2.9	30.5 ± 3.3

Percent session time is shown.

*p < 0.05 for between-state comparison (Bonferroni corrected).

To summarize, the LC phasic activation shortly preceding the presentation of a salient sound resulted in the attenuated behavioral response. The EEG modulation induced by the LC stimulation was indicative of a transient increase of cortical arousal level, which could account for the ASR reduction. In contrast to tonic LC stimulation, a brief LC activation did not cause the behavioral state change. Our results point to the LC as a part of the ASR controlling network. The LC may directly modulate the ASR brainstem circuit via its descending projections and indirectly via its diffuse ascending projections to the brain regions comprising a saliency network.

### Effect of arousal on the acoustic startle response and prepulse inhibition

**Figure 4:**
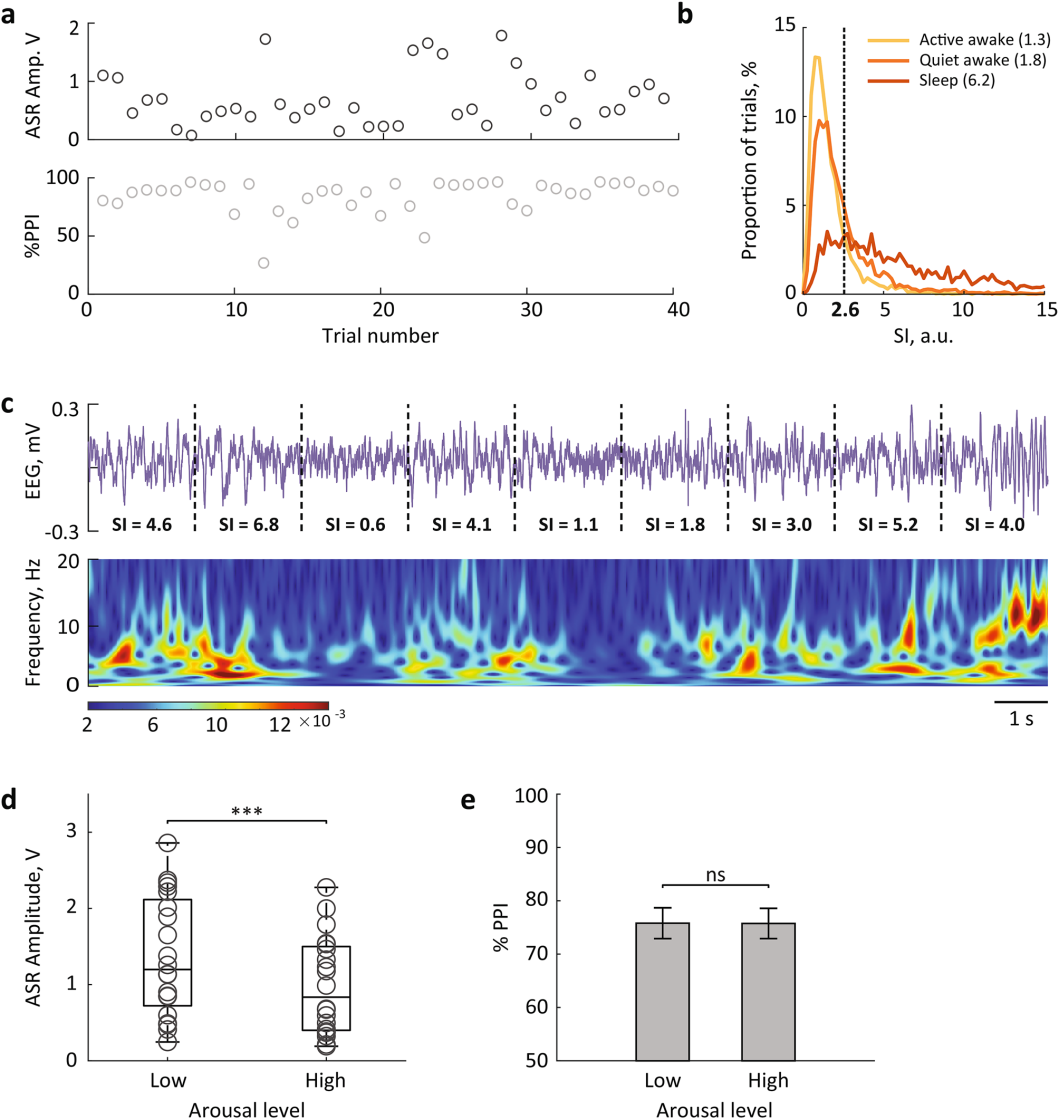
The ASR amplitude varies with arousal. (**a**) Trial-to-trial variability of the ASR amplitude (top) and %PPI (bottom) from a representative session. (**b**) Distribution and medians of SI-values during active awake, quiet awake and sleep states. The threshold for sorting the low and high arousal trials was chosen as the intersection between the SI distributions. (**c**) A representative EEG trace (top) and corresponding spectrogram (bottom) show the moment-to-moment fluctuation of the cortical arousal level; SI-value is indicated for each 2-s interval. (**d**,**e**) The ASR amplitude (**d**), but not %PPI (**e**) varied with arousal level. ***p < 0.001 (Wilcoxon signed-rank test).

To directly examine the dependency of the ASR and PPI on the arousal level, we tested additional 7 rats on the acoustic trials while simultaneously monitored the frontal EEG; no microstimulation was applied in this group (Table 1). The prepulse, startle, and prepulse paired with startle were randomly presented to the spontaneously behaving rats. Each session contained 40 to 80 repetitions of each trial type. The data were collected from 20 sessions (2–4 sessions per rat). The ASR amplitude and % PPI substantially varied across trials (Fig. 4a). As we described above, the LC phasic activation reduced the ASR and increased the cortical arousal level. We assumed that a moment-to-moment fluctuation of the arousal level could be a variability source for the startle reactivity.

To characterize the cortical arousal level, we calculated the EEG synchronization index (SI). The EEG spectral analysis is broadly used for classifying the patterns of cortical activity associated with different levels of vigilance or different sleep stages^37^. The EEG-based methods have been validated by intracellular recordings^38^. It has been documented that during NREM sleep, the membrane potentials of cortical neurons fluctuate synchronously, which produces high-amplitude slow EEG rhythms; during awake, the membrane potential of cortical neurons is maintained at a depolarized level permitting non-synchronized neuronal firing, which in turn produces low-amplitude fast EEG oscillations^39^. The cortical arousal level is commonly assessed by the degree of synchronization of the cortical population. For example, Curto and colleagues^40^ used a power ratio between 0–5 Hz and 0–50 Hz bands. In our previous work, we used a Down-State-Ratio or a band-limited power ratio for characterizing the ongoing cortical or behavioral state^35,36^. To calculate the SI, we extracted a delta (1–4 Hz)/gamma (30–90 Hz) power ratio over a 2-s time window before the stimulus onset. The SI-values varied from 0.1 to 28.2 and reflected moment-to-moment fluctuations of the cortical state (Fig. 4b,c). The distribution of SI-values for different behavioral states showed that SI < 2.6 indicated a high arousal state (active and quiet awake), while SI > 2.6 was indicative for NREM sleep (Fig. 4b). The SI did not discriminate the awake state with and without locomotion. Notably, a small proportion of NREM-episodes had low SI values, possibly indicating microarousal episodes. We split the low- and high-arousal trials according to the SI values and compared the behavioral variables. The ASR amplitude strongly depended on the arousal level (Wilcoxon signed-rank test, Z = 3.6, p = 0.0003, r = 0.8; Fig. 4d). At the same time, prepulse effectively attenuated the ASR regardless of the arousal level (Table 3). There was no difference in the efficiency of sensorimotor gating, as indicated by %PPI (Wilcoxon signed-rank test, Z = 0.1, p = 0.9, r = 0.02; Fig. 4e).

**Table 3:** The prepulse sound attenuated the ASR at both low and high arousal levels.

Max floor deflection, volts	SI-high	SI-low
Startle trials	0.8 ± 0.2	1.2 ± 0.2
Prepulse + startle trials	0.1 ± 0.04***	0.2 ± 0.06***

***p < 0.001 for comparisons between different trial types (Wilcoxon signed-rank test).

**Figure 5:**
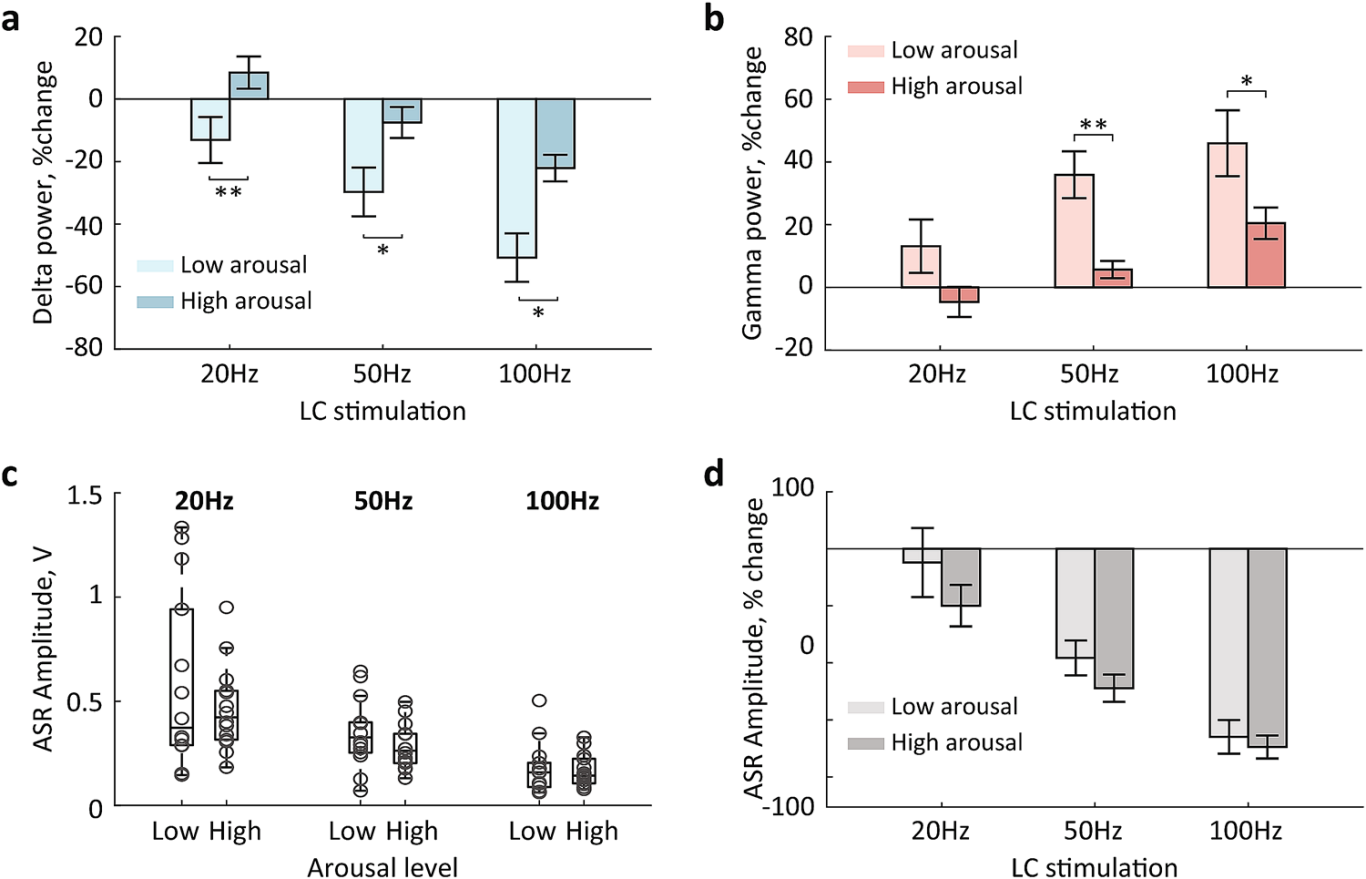
The effects of LC stimulation on the EEG and ASR. (**a**,**b**) The EEG delta (**a**) and gamma (**b**) power change produced by LC stimulation differed across arousal levels. (**c**,**d**) The ASR amplitude (**c**) and degree of ASR suppression (**d**) by preceding LC stimulation did not differ across arousal levels. *p < 0.05 and **p < 0.01 (Wilcoxon signed-rank test).

We next examined whether the effect of LC stimulation depended on the ongoing cortical state. We first quantified the EEG modulation induced by LC stimulation. We extracted the EEG delta and gamma power change (post- vs. pre-stimulation, see “Materials and methods” for details). The two-way ANOVA with the stimulation frequency (20, 50, 100 Hz) and arousal level (low vs. high) as repeated factors revealed that the EEG change depended on the stimulation frequency (delta: F_2, 26_ = 19.8, p < 0.0001, eta^2^ = 0.6; gamma: F_2.26_ = 10.1, p = 0.001, eta^2^ = 0.4) and the arousal level (delta: F_1,13_ = 17.9, p = 0.001, eta^2^ = 0.6; gamma: F_1,13_ = 10.3, p = 0.007, eta^2^ = 0.4). There was no significant frequency × arousal interaction for either delta (F_2,26_ = 0.3, p = 0.7, eta^2^ = 0.03) or gamma (F_2,26_ = 0.9, p = 0.4, eta^2^ = 0.07) power change. Thus, the EEG modulation in both delta and gamma ranges scaled with the stimulation frequency and was stronger during low arousal trials (Fig. 5a,b). Figure 5c shows the ASR amplitude for different stimulation frequencies and arousal levels. The repeated-measures ANOVA revealed the main effect of the stimulation frequency (F_1.1,14.1_ = 20.0, p = 0.0004, eta^2^ = 0.6), but not the arousal level (F_1,13_ = 1.7, p = 0.2, eta^2^ = 0.1). There was no significant frequency x arousal interaction (F_1.1, 14.6_ = 1.5, p = 0.2, eta^2^ = 0.1). We also compared the magnitude of the ASR reduction calculated as a percentage change from the ASR amplitude elicited by startle only across stimulation frequencies and arousal levels (Fig. 5d). This analysis confirmed the effect of the stimulation frequency (F_1.3,16.6_ = 26.5, p < 0.0001, eta^2^ = 0.7), but no effect of the arousal level (F_1,13_ = 2.1, p = 0.2, eta^2^ = 0.14). There was no significant frequency x arousal interaction (F_1.4,18.2_ = 0.8, p = 0.4, eta^2^ = 0.06).

Thus, both the EEG modulation and ASR attenuation produced by LC stimulation were proportional to the stimulation frequency. The neural response, as reflected by the EEG change, was also dependent on the ongoing cortical state. In contrast, both sensory gating (%PPI) and the effect of LC phasic activation on the ASR did not depend on the cortical arousal level.

### Effect of arousal on the auditory evoked potential

**Figure 6:**
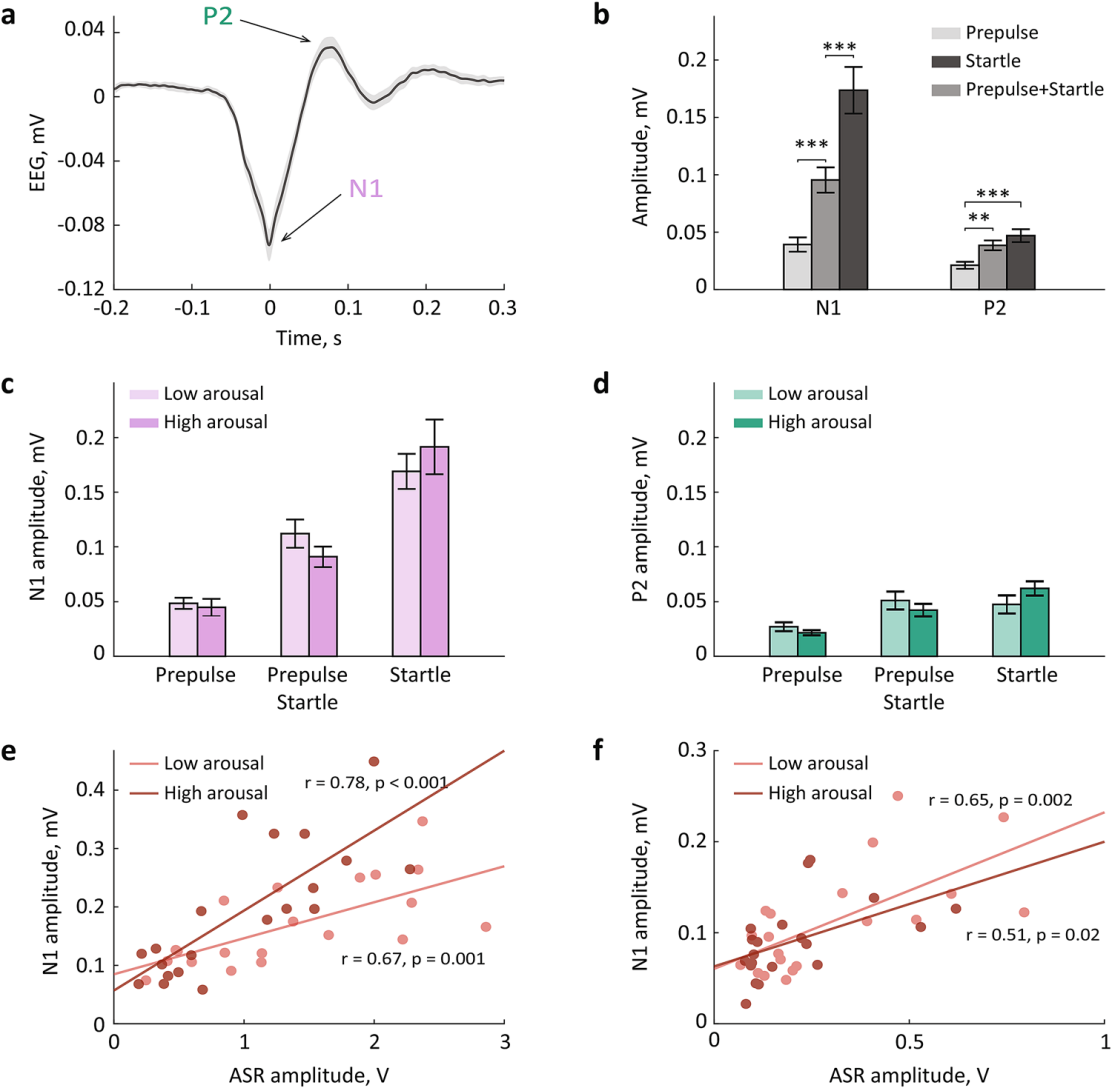
The auditory evoked potential varied across trial types, but not arousal levels. (**a**) A representative AEP trace. The peri-stimulus EEG was averaged over all startle only trials (n = 20 sessions, n = 7 rats); shadow shows s.e.m. Arrows point to the N1 and P2 peaks. (**b**) The N1- and P2-amplitude for different trial types. (**c**,**d**) The startle-elicited N1 (**c**) and P2 (**d**) amplitudes for low and high arousal trials. (**e**,**f**) The N1/ASR correlation for startle alone trials (**e**) and the trials with paired tones (**f**). **p < 0.01 and ***p < 0.001 (post hoc comparisons, Bonferroni corrected).

We sought to examine if an Auditory Evoked Potential (AEP) varies with arousal. The AEPs were readily detected in the frontal EEG. Figure 6a shows a typical AEP shape with the characteristic N1 and P2 peaks. The maximal N1 and P2 amplitude were extracted, sorted according to the trial type (prepulse, startle, startle coupled with prepulse) and arousal level (low vs high). The N1- and P2-amplitudes were submitted to a two-way repeated-measures ANOVA with 3 trial types (prepulse, startle, startle coupled with prepulse) and two arousal levels as repeated factors. The ANOVA revealed a significant effect of the trial type for both N1 (F_1.5, 27.8_ = 50.2, p < 0.0001, eta^2^ = 0.7) and P2 (F_2,38_ = 15.1, p < 0.0001, eta^2^ = 0.4) amplitude, but no significant effect of arousal (N1: F_1,19_ = 0.02, p = 0.9, eta^2^ = 0.001; P2: F_1,19_ = 0.001, p = 0.97, eta^2^ < 0.0001). There was a significant trial type × arousal interaction for the N1-amplitude (F_1.5, 28.1_ = 4.1, p = 0.04, eta^2^ = 0.2), but not the P2-amplitude (F_1.4, 27.3_ = 2.2, p = 0.1, eta^2^ = 0.1). As expected, both N1- and P2-amplitude increased with higher sound intensity (Fig. 6b). Moreover, the N1-amplitude was lower when the startling sound was preceded by prepulse, just as the behavioral response (Fig. 2b). We observed no arousal-dependent effects on N1- and P2-amplitude (Fig. 6c,d). There was a tendency of a lower N1-amplitude for high arousal trials, but only for the trials with paired tones (Wilcoxon signed-rank test, Z = 1.8, p = 0.07, r = 0.4).

Finally, we assessed the relationships between the behavioral (ASR) and neuronal (AEP) response to acoustic stimulation by correlating the ASR and N1/P2 amplitudes. Since the prepulse tone did not elicit any reliable ASR, we included the startle trials and trials with paired tones in this analysis. We found systematic relationships between the ASR and N1, but not P2 (startle tone: r = 0.3, p = 0.2; paired tones: r = 0.4, p = 0.1). The ASR/N1 correlation was highly significant for each trial type (startle: r = 0.8, p < 0.0001; paired: r = 0.6, p = 0.01), but the correlation strength did not differ across arousal levels (Steiger’s Z: startle: Z = − 1.2, p = 0.1; paired: Z = 0.9, p = 0.2; Fig. 6e,f). Neither N1, nor P2 correlated with %PPI (N1: r = 0.3, p = 0.2; P2: r = 0.1, p = 0.6; not shown). Thus, both N1- and P2-amplitude reflected the auditory stimulus intensity, but only N1-amplitude was indicative of the behavioral responsivity to the auditory stimulation. The N1/ASR relationships, however, were not arousal-dependent.

Overall, our results are consistent with a view that activation of the arousal network underlies the ASR attenuation. We provided here several lines of evidence supporting this view. First, we have demonstrated that phasic LC activation reduced the ASR. Second, the LC stimulation rapidly elicited transient cortical arousal (as reflected by EEG delta and gamma power change). Third, we showed that a state of higher cortical arousal was associated with a weaker behavioral response. Our finding that the LC activation effectively modulated the ASR during states of high and low cortical arousal suggests that the LC may present a critical brainstem component regulating the ASR circuit.

## Discussion

Our present study demonstrated that a brief phasic LC activation shortly preceding a startle-eliciting sound reduced the magnitude of the ASR in spontaneously behaving rats. The behavioral effect scaled with the frequency of LC stimulation and at 100 Hz mimicked the acoustic prepulse. The ASR attenuation by preceding LC activation was accompanied by a change of the EEG spectral components that were indicative of higher cortical arousal. In the microstimulation-free condition, the ASR amplitude was the lowest during the high arousal state. Thus, the effect of LC stimulation on the ASR may be mediated by activation of the arousal network; however, multiple alternative mechanisms including the LC directly affecting the brainstem ASR circuit, auditory input, or motor output cannot be ruled out.

A long-standing view attributed the ASR attenuation by prepulse to inhibition of the acoustically responsive PnC neurons by cholinergic input from the PPTg and the laterodorsal tegmentum (LDTg)^3^. Indeed, a priming stimulation of the PPTg or LDTg reduced the ASR^41^. However, the evidence is accumulating that the mechanism regulating the ASR/PPI circuit is more complex than previously thought. For example, it has been reported that knockout mice with reduced cholinergic tone had normal ASR and PPI^13^. Another study has shown that selective cholinergic lesions in the PPTg reduced the ASR without affecting the PPI^12^. Most recently, it has been documented that optogenetic activation of the PPTg cholinergic neurons enhanced the ASR^14^. Thus, GABAergic and/or glutamatergic PPTg neurons appear to be involved in the regulation of excitability within the ASR/PPI circuit^9^. Besides, it has been previously suggested that the role of cholinergic inputs to the ASR/PPI circuit may be restricted to the modulation of arousal^9^. Arousal mediating mechanism may underlie the effects of LC activation on the ASR.

The LC-NA neurons project diffusely throughout the forebrain and may affect sensorimotor processing via modulation of arousal^15^. Previous studies in rats and humans have demonstrated that pharmacological suppression of NA neurotransmission lowered arousal level and reduced the ASR amplitude^24,42,43^, while enhanced NA neurotransmission disrupted the PPI, possibly due to hyperarousal^29^. A combination of the elevated tonic firing of LC-NA neurons with a reduced sensory-evoked LC phasic response that is associated with the states of high arousal^31,44^ may result in less efficient sensorimotor integration. Here, we reported that during spontaneous behavior, pairing the LC stimulation with a startle-eliciting sound reduced the ASR. The ASR reduction after phasic LC activation was accompanied by a rapid (~ 40 ms) EEG desynchronization, as was reflected by a power decrease in lower (delta) and increase in higher (gamma) frequencies. The ASR reduction and EEG power change were state-dependent and proportional to the LC stimulation frequency. Thus, a transient increase of cortical arousal may underlie the ASR reduction. This view is consistent with the dependence of the ASR on the cortical arousal level. We consistently observed a higher proportion of low amplitude ASRs during active awake. The same observation was reported in an earlier study in cats^45^, yet no difference across the awake/sleep cycle was found in rats^46^. In humans, the ASR amplitude is typically reduced by sedative drugs, including the ones acting on the cholinergic and noradrenergic receptors^42,43,47^. Thus, as was postulated for a higher-order cognition^48^, the sensorimotor gating appears to benefit from the optimal level of the NA neurotransmission that is largely provided by a balanced tonic/phasic firing of the LC-NA neurons.

If the LC stimulation in our experiments mimicked the acoustic prepulse, regardless of the exact modulation target, LC neurons are expected to respond to acoustic stimuli of prepulse intensity. The LC auditory response is known to be highly dependent on sound parameters and behavioral context. Earlier studies reported that sounds of moderate volume (70 dB, 32 ms) did not elicit any LC response^49^, while sounds eliciting an orienting response (> 96 dB) were effective^17,18,49,50^. In monkeys, LC response to a 70 ms burst of white noise at 75 dB was stronger during drowsiness than during cognitive task performance^31^. A recent study in rats demonstrated a robust LC response to 1 s tones at 76 dB^51^, but LC response to shorter (0.25 s) sounds at 74 dB appears less reliable, as only 14% of neurons were responsive^52^. Although LC responses to short (20–40-ms) sounds, like the ones used in the ASR/PPI, remain to be characterized, existing evidence indicates that the LC may respond to prepulse. The latency of LC auditory response of 20–24 ms^18,53^ is in good agreement with a delay of 20–500 ms between the prepulse and startle stimuli used in the PPI paradigm. It has been also reported that low-intensity sounds (~ 70 dB) reliably elicit a large-amplitude deflection of the extracellular potential in the LC with no accompanying motor response^53^. This sound-evoked potential resembles a ponto-geniculo-occipital (PGO) wave, a hallmark of REM sleep. The PGO-like waves occur outside REM sleep and indicate activation of the reticular alerting network^54,55^. Our observation that outside-LC stimulation failed to induce the EEG desynchronization was consistent with the absence of PGO-like waves in the pontine regions adjacent to the LC^53^. Thus, non-startling prepulse-like sounds induce enhanced peri-synaptic activity in the LC, while startling sounds evoke the LC phasic response and drive selection of adaptive behavior. The LC phasic photoactivation produced attentional signals, such as the P300 event-related potential, within the sensory processing network, including “false salience” in the absence of intense stimulus^30^.

In our experiments, to elicit a synchronous discharge of LC neurons, we applied a mild electric current (0.05 mA) in direct proximity to the LC cell bodies. In our earlier study, we used similar stimulation parameters and demonstrated that unilateral current application elicits a robust discharge of LC neurons bilaterally; we also showed that stimulation-induced biphasic response profile (excitation followed by inhibition) resembled a naturalistic LC response to salient stimuli (e.g. foot shock)^32^. Moreover, the current intensity of 0.05 mA did not cause any neuronal damage around the electrode tip and the current spread did not exceed the size of the LC core^32^. Most importantly, in behaving rats, identical LC stimulation produced a transient change in the forebrain activity without causing awakening from natural sleep^35^. Although individual LC neurons typically fire at rates below 5 Hz, brief trains of pulses at 20–100 Hz possibly mimicked a synchronous discharge of the LC neuronal population; a burst-like discharge, as well as phasic stimulation, is more efficient for NA release^56,57^. Leaving aside a debatable cell-type selectivity of microstimulation, phasic LC activation was sufficient for increasing EEG arousal without causing awakening or any abrupt change of ongoing behavior. The LC stimulation at 20 Hz was not sufficient for the significant ASR reduction and caused no (or weak) EEG desynchronization. In contrast, the high-frequency (100 Hz) LC stimulation caused a pronounced change in the EEG and the magnitude of the ASR attenuation was comparable with the one produced by the auditory prepulse. Notably, the modulation of sensorimotor gating by LC phasic activation did not depend on the arousal level. We believe that in parallel to the effects in the forebrain, the LC stimulation caused a generalized reticular activation leading to a reduced ASR. The reticular formation being a site of NA action may explain a similar degree of the ASR modulation across different arousal levels.

Apart from activating the arousal network, the LC can affect the ASR/PPI circuit through modulating sensory input or auditory perception. The LC directly projects to the CRN^58^ and other subcortical and cortical structures within the primary auditory pathway^59^. Pairing auditory stimulation with NA release leads to increased neuronal excitability and responsiveness in the auditory thalamus and cortex^22,23^. The LC projects to the IC, which is activated by the acoustic prepulse and exerts an inhibitory influence on the primary ASR pathway^41^. The IC receives up to 97% of its noradrenergic innervation from the LC^59, 60^ and NA release associated with LC activation may enhance the IC neuron excitability. The direct electrical stimulation of the IC attenuated the ASR^33^. Moreover, NA-mediated cortical arousal may engage cholinergic input to the IC via auditory cortex projections to the PPTg and LDTg^61^. Altogether, NA release within the auditory network may lead to a change in the auditory signal processing and perception. In our study, priming LC stimulation may have weakened the perception of the sound volume. In parallel, the LC may modulate the ASR/PPI circuit through its forebrain projections to the hippocampus, amygdala, or prefrontal cortex^62^. A long-standing view on the mechanism underlying the inhibitory effect of prepulse on the ASR considers the attentional shift towards a sudden sensory input^63^. Such an attentional shift may trigger reorienting and change of ongoing behavior^1^. The LC role in promoting attentional shift and reorienting is well known^16^. The orienting response toward a new stimulus may also inhibit the startle reflex^41^. Lastly, the LC can modulate motor outflow through its direct projections to the spinal motor neurons^64^. To the best of our knowledge, the role of the coeruleospinal pathway for modulation of the ASR/PPI circuit remains unclear. An earlier study showed that intrathecal administration of clonidine suppressed the ASR^25^. Phasic LC activation could reduce the excitability of spinal motor neurons and therefore the ASR magnitude via presynaptic inhibition^65^. The potentiation by NA of glycine-mediated inhibition in the spinal dorsal horn neurons has been also described^66^.

In the present study, we also examined the effect of arousal on the AEPs recorded in the frontal EEG. The AEPs are recorded in many subcortical and cortical structures. The N1 and P2 peaks, two early AEP components, are commonly considered to reflect the sound intensity^67–69^. The AEP amplitude, like other sensory responses, is attenuated during low arousal states^70–72^. The state-dependency of AEPs greatly depends on their origin. The AEPs recorded from the rat primary auditory cortex were not modulated by vigilance state^73^. The EEG study in rats showed that the AEP amplitude in the frontal and parietal, but not in the occipital areas varied with arousal^70^. The AEPs originating from the reticular ascending inputs are typically state-dependent and rapidly habituate^74^. In the rat frontal EEG, we observed sound- but not  arousal-modulated AEP. Differential AEP modulation was also observed in humans. In the frontal and central EEG, the N1 amplitude elicited by a low volume sound (~ 30 dB) decreased in lower arousal state^71^, but N1 elicited by louder tones (~ 75 dB) was higher^75^. The inconsistent evidence for arousal-modulated AEP further illustrates that the state-dependency of the auditory processing depends on the engaged network and the features of sensory input. This notion is in agreement with previous reports that drugs disrupting the PPI may not affect the AEPs^47,76^. Finally, we revealed that the ASR and N1 amplitudes were correlated; thus, the N1 component of AEP may reflect both sensory input and motor output. Since the ASR/PPI tests are used for diagnostics, the event-related EEG oscillations, including the AEP, could provide a complementary tool to study psychopathology in clinical practice.

All in all, clearly there are multiple neural pathways, which underlie a rather complex regulation of a seemingly simple acoustic startle reflex. Different aspects of sensorimotor processing are reflected by various neural representations. The behavioral and neural correlates of sensorimotor processing may not share the same mechanism. Overall, our findings are consistent with a view that the startle reflex depends on the state of the organism. Our results suggest the involvement of the LC-NA system in the modulation of the ASR/PPI circuit, possibly via affecting the arousal network. The LC connectivity in the brainstem also supports the LC direct influencing the primary ASR/PPI circuit. The exact mechanisms underlying the effects of LC phasic activation on sensorimotor gating described here could and should be established in future studies.

## Materials and methods

### Animals

Twenty-one adult male Sprague–Dawley rats (Charles River Laboratory, Germany) weighing 300–450 g were used. After surgery rats were single-housed and had access to food and water ad libitum. Animals were tested between 10 a.m. and 6 p.m. during the dark phase of a 12 h light/dark cycle (8 a.m. lights off). All experiments were conducted following the German Animal Welfare Act (TierSchG) and Animal Welfare Laboratory Animal Ordinance (TierSchVersV). This is in full compliance with the guidelines of the EU Directive on the protection of animals used for scientific purposes (2010/63/EU). The study was reviewed by the ethics commission (§15 TierSchG) and approved by the state authority (Regierungspräsidium, Tübingen, Baden-Württemberg, Germany).

### Surgery and electrode placement

Animals were anesthetized with isoflurane (initiation 4%, maintenance 1.5–2.0%). The depth of anesthesia was controlled by ensuring a lack of responses to mildly noxious stimuli (a hind paw pinch). Heart rate and blood oxygenation were monitored using a pulse oximeter (Nonin 8600V, Nonin Medical, Inc., Plymouth, MN); supplementary oxygen was provided to maintain the blood oxygenation level above 90%. Body temperature was maintained at ~ 37 °C throughout the entire anesthesia period. A fully anesthetized rat was fixed in a stereotaxic frame; the skull surface was adjusted horizontally. The skull was exposed and local anesthetic (Lidocard 2%, B. Braun, Germany) was applied on the skin edges to additionally numb the skin. Burr holes were made for electrodes and anchor screws. For EEG recording, a stainless steel screw (0.86 mm diameter, FST, Germany) was placed above the frontal cortex and the ground screw was placed above the cerebellum. Four anchor screws (1.19 mm diameter, FST, Germany) were placed on the skull side edges. Screws were fixed in the skull and additionally secured with tissue adhesive. The stimulation electrode (single platinum-iridium electrode, FHC, Bowdoin, ME) was placed in the LC using a high-precision stereotaxic micromanipulator (David Kopf Instruments, Tujunga, CA). The monopolar stimulation electrode was implanted at 15° angle 4.0–4.2 mm posterior to lambda, 1.0–1.2 mm lateral, and 5.5–6.2 mm deep. The accuracy of LC targeting was verified by online monitoring of neural activity. The LC neurons were identified by broad spike widths (~ 0.6 ms), regular low firing rate (1–2 spikes/s), and biphasic (excitation followed by inhibition) response to paw pinch. Once the electrode depth was optimized, the entire implant was secured on the skull with dental cement (Paladur, Heraeus Kulzer GmbH, Germany). The injection of analgesic (2.5 mg/kg, s.c; Finadyne, Essex) and antibiotic (5.0 mg/kg, s.c.; Baytril, Bayer) was given before rat awakening from anesthesia and repeated at 24 h intervals for 4 days. Animals were allowed 1 week of post-surgery recovery.

### Electrophysiological recording and electrical stimulation

We used the same data acquisition setup as described elsewhere^35^. The EEG and ground electrodes were connected to the multichannel amplifier (MPC Plus, Alpha Omega Engineering, Israel) through an analog headstage (Plexon Inc, Dallas, USA), a flexible cable (Plexon Inc, Dallas, USA), and an in-house built preamplifier. The EEG signal was filtered (0.1–300 Hz), amplified (× 1k), and digitized using Power1401mkll (CED, UK). The stimulation and ground electrodes were connected to an in-house built current source via self-made cable via a 6-channel electrode pedestal (P1 Technologies, Roanoke, USA). The Spike2 software (CED, UK) and a digital-to-analog converter (Power 1401mkII, CED, UK) were used for controlling the current parameters. The voltage passed through the electrode tip was monitored via a custom-designed voltage output unit. The 100-ms trains of biphasic (cathodal leading) square pulses (0.4 ms, 0.05 mA) were delivered unilaterally at 20, 50, and 100 Hz. The stimulation parameters were selected based on our previous studies that characterized in detail the local and distal effects of the LC electrical stimulation^32,35^. Briefly, it has been shown that the current intensity of 0.05 mA does not cause neuronal damage around the electrode tip and the spread of depolarizing current does not exceed the size of the LC core. The unilateral current application elicited a robust discharge of LC neurons bilaterally and stimulation-induced discharge resembled a naturalistic LC response to salient stimuli. The LC stimulation at frequencies above 50 Hz induced a transient change in the forebrain activity without causing awakening from natural sleep. Before the main experiment, each rat was submitted to test stimulation when the stimulation effectiveness and the stimulation parameters were calibrated. The parameters of LC stimulation were selected such that the strongest stimulation induced a transient change in the EEG power spectrum without causing awakening from sleep or any adverse behaviors.

### Behavioral testing

Rats were first habituated to a sound-attenuated chamber (60 cm × 40 cm × 40 cm) and the cable plugging procedure. After habituation, rats were tested on acoustic, microstimulation, and mixed (microstimulation/acoustic) trials as shown in Fig. 1b. The acoustic trials included startle (broadband noise, 40 ms, 100/105 dB), prepulse (10 kHz, 20 ms, 70/75 dB), or prepulse followed by startle with a 100-ms delay. The microstimulation trials included 100-ms trains of pulses at 20, 50, and 100 Hz. Mixed trials included microstimulation immediately followed by startle. Each trial type was randomly presented and repeated 40–80 times. The ITI varied between 10 and 20 s to avoid animal habituation to sounds. The trial presentation was controlled using the Spike2 software (CED, UK). Each session started with a 5-min habituation period, during which a continuous white background noise (50/55 dB) was presented. Each rat was tested in 1 to 5 sessions. The movement of the animal was measured via four floor-mounted vibration sensors; the floor deflection amplitude was converted to voltage and synchronized with EEG recording. The maximal movement amplitude was extracted from the 500-ms window after stimulus presentation. Baseline movement activity was calculated as maximal amplitude in a 1-s time window before the sound onset. The PPI was quantified as following: %PPI = (1 – ASR _prepulse + startle_/ASR_startle_) * 100%.

### EEG spectral analysis

The effectiveness of LC stimulation was measured as a change in the EEG delta (1–4 Hz) and gamma (60–90 Hz) power. We used a high-gamma range to avoid EEG contamination with the artifacts produced by electric pulses at 50 Hz. The EEG delta oscillations are predominant during a low arousal state, such as NREM sleep and delta power fluctuates with arousal^77^. The EEG gamma activity is commonly interpreted as a signature of cortical and/or behavioral arousal^78,79^. Modulation of gamma oscillations has been implicated in saliency processing^80–82^. Based on extensive evidence, we used the EEG delta and gamma power change as a signature of cortical arousal. To quantify the EEG change, we extracted the band-limited power—1-s to 3-s around the stimulation onset using a multi-taper method (http://chronux.org/)^83^. The power was z-score normalized to a 1-s window before the stimulation onset and averaged across trials (20, 50, and 100 Hz). The power change above 1.96 z-score was considered as significant. To characterize the magnitude of the band-limited power change, we computed the power spectrum around LC stimulation; the 100-ms stimulation interval was excluded due to artifacts. The time window of ± 1 s and ± 0.2 s was used for the delta and gamma band, respectively. We then calculated the power change as a percentage of the pre-stimulation level.

To classify the behavioral state, each 2.5-s recording epoch was assigned to active awake, quiet awake, or NREM sleep using EEG and the movement detector. The epochs of active awake were identified by the presence of active locomotion; the epochs of quiet awake were identified by the absence of motor activity and above threshold theta (6–10 Hz)/delta (1–4 Hz) ratio; the epochs of NREM sleep were identified by the absence of motor activity and below threshold theta/delta ratio. The minimal duration of the same behavioral state was set to 20 s. The epochs in which the behavioral state could not be classified were excluded from the analysis (3.16 ± 0.25% of total recording time).

To quantify the cortical arousal level immediately preceding the stimulus presentation, we used a Synchronization Index (SI). The SI was calculated over a 2-s time window before the stimulus onset as a power ratio between delta (1–4 Hz) and gamma (30–90 Hz) bands. The SI distributions were calculated for each behavioral state and the SI-value at the intersection between distributions was used as a threshold for sorting the low and high arousal trials (Fig. 4b).

### Statistical analysis

The ASR amplitude distributions from different trial types were compared using the Kolmogorov–Smirnov test. The non-parametric Wilcoxon signed-rank test was used for paired-comparisons of the population mean rank. The effect size of the Wilcoxon signed-rank test was calculated as $=\frac{Z}{\sqrt{Npair}}$ . Different designs of analysis of variance (ANOVA) were used for comparing experimental conditions; the Greenhouse–Geisser correction was applied when the sphericity assumption was violated. The Bonferroni test was used for post-hoc comparisons. Pearson’s linear correlation was used for correlation analysis. Steiger’s Z test was used to compare the correlation strength. The statistical significance (α-value) was set at p = 0.05. The IBM SPSS Statistics (v.22) and Matlab (MathWorks) software packages were used for statistical analysis.

### Perfusion and histology

After the final recording session, rats were euthanized (100 mg/kg, i.p., Narcoren, Merial) and perfused. Brains were removed and stored in paraformaldehyde until used. Before sectioning, brains were impregnated with sucrose until they sank. Serial 60-µm-thick coronal sections were cut on a horizontal freezing microtome (Microm HM 440E, Walldorf, Germany) and then directly Nissl stained or stored at − 20 °C in a cryoprotectant solution until further processing. All sections were examined using an AxioPhot or AxioImager microscope (Carl Zeiss, Goettingen, Germany). The electrode tracks were localized visually and digitized; the placement of the electrode tip was reconstructed.
